# Perceived disabling physical pain and suicidal ideation in aging people living with HIV cured of hepatitis C: A multi‐center survey in France (ANRS CO13 HEPAVIH)

**DOI:** 10.1111/hiv.70121

**Published:** 2025-10-01

**Authors:** Tangui Barré, Clémence Ramier, Camelia Protopopescu, Philippe Sogni, Karine Ory, Tounes Saidi, Sophie Abgrall, Sylvie Brégigeon‐Ronot, Patrizia Carrieri, Fabienne Marcellin, the ANRS CO13 HEPAVIH Study Group, D. Salmon, L. Wittkop, P. Sogni, P. Carrieri, B. Spire, K. Ory, P. Trimoulet, J. Izopet, L. Serfaty, L. Alric, M. A. Valantin, G. Pialoux, J. Chas, K. Barange, A. Naqvi, E. Rosenthal, A. Bicart‐See, O. Bouchaud, A. Gervais, C. Lascoux‐Combe, C. Goujard, K. Lacombe, C. Duvivier, D. Neau, P. Morlat, F. Bani‐Sadr, F. Boufassa, C. Solas, H. Fontaine, L. Piroth, A. Simon, D. Zucman, F. Boué, P. Miailhes, E. Billaud, H. Aumaître, D. Rey, G. Peytavin, O. Zaeglel‐Faucher, D. Salmon, R. Usubillaga, P. Sogni, B. Bienvenu, O. Launay‐Puybasset, C. Bernasconi, A. Brunet, B. Silbermann, H. Boucher, F. Rollot, O. Zak Dit Zbar, S. Pol, C. Le Jeunne‐Ballif, P. Louergue, H. Mehawej, L. Merine‐Belardi, F. Almasi, N. Benammar, L. Alagna, S. Chaussade, L. Guillevin, B. Terris, C. Norgeux, C. Katlama, M. A. Valantin, H. Stitou, F. Caby, L. Paris, L. Schneider, R. Tubiana, A. Simon, L. Roudière, O. Benveniste, G. Breton, M. Bonmarchand, M. Kirstetter, F. Charlotte, F. Capron, V. Calvez, V. Thibaut, C. Soulie, B. Abdi, S. Lambert‐Niclot, S. Bregigeon‐Ronot, O. Zaegel‐Faucher, H. Laroche, A. Menard, E. Gaubert‐Marechal, S. Thirée, C. Tamalet, C. Hakimi, J. C. Poluzzi, C. Solas, G. Pialoux, J. Chas, L. Lassel, T. Lyavanc, S. Le Nagat, L. Slama, A. Canesti, P. Callard, B. Bachour, L. Alric, N. Sigur, D. Bonnet, M. Guivarch, K. Barange, J. M. Peron, P. Massip, L. Cuzin, M. Obada, E. Bonnet, M. Alvarez, E. Labau, M. Mularczyk, N. Bourdoncle, J. Penecy, L. Porte, F. Busato, S. Khatibi, J. Bernard, J. Selves, F. Larroquette, V. Ferrer, J. Chiabrandon, E. Rosenthal, C. Pradier, V. Mondain, P. Dellamonica, P. M. Roger, S. Ferrando, C. Ceppi, A. Naqvi, V. Rio, B. Dunais, B. Prouvost, F. De Salvadore‐Guillouet, I. Prebost, J. Durant, P. Pugliese, J. F. Michels, M. C. Saint‐Paul, C. Partouche, K. Nerini, O. Bouchaud, S. Abgrall, I. Zamord, F. Bidegain, M. Azghay, C. Rouyer, M. Tatay, F. Mechai, A. Martin, Y. Baazia, V. Iwaka‐Bande, A. Gerber, A. Bicart‐See, D. Garipuy, M. J. Ferro‐Collados, F. Gaches, F. Abravanel, F. Larroquette, J. Chiabrandon, A. Gervais, Y. Yazdanpanah, D. Henin, M. Bertine, M. Onambele, G. Peytavin, C. Lascoux‐Combe, T. Kandel, C. Pintado, J. M. Molina, C. Gatey, S. Gallien, W. Rozenbaum, M. Lafaurie, A. L. Munier, D. Ponscarme, M. Previllon, N. Colin De Verdière, M. G. Tateo, P. Palmer, A. Gabassi, R. Amarsy, M. C. Mazeron, K. Lacombe, J. Krause, D. Wendum, D. Fofana, C. Goujard, Y. Quertainmont, E. Teicher, L. Escaut, O. Derradji, C. Pallier, F. Veillet, L. Mouna, O. Lortholary, C. Duvivier, C. Rouzaud, M. Shoai Tehrani, S. Boucly, G. Obenga, J. P. Viard, E. Gardiennet, A. Mélard, D. Neau, A. Ochoa, E. Blanchard, C. Cazanave, M. Dupon, H. Dutronc, F. Dauchy, H. Wille, P. Bioulac‐Sage, P. Trimoulet, P. Morlat, D. Lacoste, F. Bonnet, N. Bernard, M. Hessamfar, F. Paccalin, C. Martell, M. C. Pertusa, M. Vandenhende, P. Mercié, T. Pistone, M. C. Receveur, M. Méchain, P. Duffau, C. Rivoisy, I. Faure, D. Malvy, J. Roger‐Schmeltz, D. Dondia, P. Bioulac‐Sage, P. Trimoulet, J. L. Pellegrin, E. Lazzaro, P. Bioulac‐Sage, P. Trimoulet, D. Zucman, C. Majerholc, J. E. Kahn, S. Hilaire, M. Brollo, F. Boué, S. Abgrall, J. Polo Devoto, I. Kansau, V. Chambrin, C. Pignon, C. Gatey, M. Favier, C. Deback, C. Vauloup, C. Pailler, F. Veillet, L. Mouna, J. L. Lopez Zaragoza, Y. Lévy, J. D. Lelièvre, A. S. Lascaux, S. Scerra, M. Bouvier‐Alias, E. Billaud, F. Raffi, C. Allavena, V. Reliquet, D. Boutoille, C. Biron, M. Lefebvre, N. Hall, S. Bouchez, C. Bernaud, O. Mounoury, A. Rodallec, L. Le Guen, P. Miailhes, D. Peyramond, C. Chidiac, F. Ader, F. Biron, A. Boibieux, L. Cotte, T. Ferry, T. Perpoint, M. Amiri, F. Valour, J. Koffi, F. Zoulim, F. Bailly, P. Lack, M. Maynard, S. Radenne, C. Augustin‐Normand, E. Vassel, T. T. Le‐Thi, L. Piroth, P. Chavanet, M. Duong Van Huyen, M. Buisson, A. Waldner‐Combernoux, S. Mahy, A. Fillion, H. Aumaître, M. Jean, A. Eden, M. Medus, M. Saada, S. Gaba, F. Bani‐Sadr, D. Lambert, Y. Nguyen, J. L. Berger, C. Rouger, V. Brodard, D Rey, M. Partasani, C. Cheneau, M. Priester, C. Bernard‐Henry, E. de Mautort, A. Ertle, F. Jegou, A. Fush, P. Gantner, S. Fafi‐Kremer, L. Pinheiro, F. Roustand, I. Kmiec, L. Traore, S. Le Puil, M. G. Tateo, S. Benothame, C. Pomes, C. Louisin, M. Mole, A. Adda, P. Thibaut, S. Poirier, R. Ben Rayana, M. P. Pietri, V. Le Baut, Y. Cuvillier, M. Alabatanah, F. Barret, F. Juster, C. Chesnel, D. Beniken, M. Sebire‐Le Cam, R. Monard, F. Badji, S. Caldato, T. Rojas Rojas, A. S. Ritleng, A. Ivanova, L. Chalal, Z. Julia, M. Cavellec, C. Chevalier, E. Paredes‐Manyari, S. Breau, N. Boughdiri, P. Fischer, C. Charles, S. Ogoudjobi, C. Brochier, V. Thoirain‐Galvan, L. Traore, S. Chaussee, S. Benothmane, A. Sondro, O. Camou, V. Godard, M. Kai, C. Gilbert, M. Pailler, L. Merchadou, L. Esterle, K. Ory, S. Gillet‐Rousseau, C. Khan, L. Abbad, M. Chalouni, V. Conte, F. Marcellin, R. Knight, M. Mora, C. Protopopescu, P. Roux, T. Barré

**Affiliations:** ^1^ Aix Marseille Univ, Inserm, IRD, SESSTIM, Sciences Economiques & Sociales de la Santé & Traitement de l'Information Médicale, ISSPAM Marseille France; ^2^ Université Paris Descartes Paris France; ^3^ INSERM U1223, Institut Pasteur Paris France; ^4^ Service d'Hépatologie, Hôpital Cochin, Assistance Publique–Hôpitaux de Paris Paris France; ^5^ Université Bordeaux, INSERM, MART, UMS 54 Bordeaux France; ^6^ Department of Clinical Research, ANRS Emerging Infectious Diseases (ANRS MIE) Paris France; ^7^ Assistance Publique‐Hôpitaux de Paris (APHP), Hôpital Béclère, Service de Médecine Interne Clamart France; ^8^ APHP, Université Paris‐Saclay, Université Paris‐Sud, UVSQ, CESP INSERM U1018 Le Kremlin‐Bicêtre France; ^9^ Clinical Immunohematology Department, Marseille Public University Hospital System (AP‐HM), Sainte‐Marguerite Hospital, Aix‐Marseille University Marseille France

**Keywords:** depression, HCV cure, HIV‐HCV co‐infection, self‐reports, suicide risk

## Abstract

**Introduction:**

Suicidal ideation (SI) is highly prevalent among people living with HIV (PWH) and those with chronic hepatitis C virus (HCV) infection. Individuals with long‐term HIV–HCV co‐infection face specific health challenges, including heightened physical pain. We aimed to assess whether disabling physical pain is associated with SI in aging PWH who have been cured of HCV, after controlling for potential correlates or confounders such as depression and psychoactive substance use.

**Methods:**

We analysed data from HCV‐cured PWH who participated in a multi‐center cross‐sectional survey embedded within the French ANRS CO13 HEPAVIH cohort. We performed a multivariable logistic regression model with SI (score >0 for the ninth item of the Patient Health Questionnaire‐9) as the outcome. Disabling physical pain was assessed using an answer ≥'very much' to the third item from the WHOQOL‐HIV BREF questionnaire.

**Results:**

Study population comprised 396 HCV‐cured PWH (73.2% male), among whom 17.7% reported SI and 11.9% reported disabling physical pain. Participants reporting disabling physical pain had a three‐fold higher risk of SI (adjusted odds ratio [95% confidence interval]: 3.07 [1.29–7.34]), after adjustment for depression (5.52 [2.66–11.43]), substance use, and lower social relationships‐related quality of life (0.72 [0.64–0.80]).

**Conclusions:**

These findings highlight that disabling physical pain should be systematically addressed among PWH cured of HCV, given its independent association with SI. Routine HIV follow‐up care should integrate systematic screening for pain, mental health problems, and lack of social support. Timely referral to specialized services may help prevent future suicidal behaviours in this population.

## INTRODUCTION

Mental health problems are highly prevalent among people living with HIV (PWH), particularly depression [1, 2], and psychoactive substance (thereafter ‘substance’) use disorders [3, 4, 5, 6]. In the first 2 years after diagnosis, PWH are at particularly high risk of depression and suicide [7]. Likewise, people with a longer history of HIV infection can also bear a high burden of depression, potentially related to the number of comorbidities they experience [8, 9]. Of particular concern, PWH exhibit higher rates of suicidal ideation (SI) and suicide than the general population [10, 11, 12, 13]. It is noteworthy that SI is highly prevalent in sub‐groups of people overrepresented in the PWH population, namely people who inject drugs and men who have sex with men [14, 15]. As a predictor of both attempted and completed suicide, SI represents a major health concern, linked to mental disorders, such as depression and anxiety [16]. It is also associated with social health determinants such as living alone or being unmarried [17, 18, 19], being the subject of gossip [18], poor family or social support [16, 20, 21, 22], and perceived stigma [21, 23, 24]. Moreover, SI has been associated with both lower adherence to anti‐retroviral therapy and higher HIV viral loads [17, 20, 25, 26]. Identifying factors associated with SI in PWH is therefore essential for improving screening and implementing timely interventions to prevent suicide attempts and optimize HIV care.

Pain is among the most frequently reported symptoms in PWH [27], with older age being a primary risk factor [28]. Given its impact on multiple dimensions of quality of life [29], PWH consistently identify pain as a key health priority [30]. Pain is associated with substance use and depression, as well as with a reduced adherence to antiretroviral therapy [31, 32]. However, few studies have examined the association between pain and SI in PWH. Although two studies have reported pain to be associated with suicidality or SI [33, 34], they did not simultaneously adjust for substance use, depression, and social factors. From a small sample of PWH from Alabama, Dafoe & Stewart reported a positive association between pain severity (using a continuous measure) and suicidality (including ideation, intent, and plan). They did not adjust their analyses for depression but included a reported ‘number of psychiatric diagnoses’ that indifferently summed up the presence of anxiety, mood, and substance use disorders [34]. In Rukundo et al.'s study from PWH in Uganda, the association found between physical pain (presence/absence) and SI or attempts was no longer significant after multivariable adjustment, including a recent HIV diagnosis [33]. Given the interrelated nature of these factors [35, 36, 37], there is a need to understand their respective contributions to SI.

Hepatitis C is known to be associated with a high prevalence of both depression [38] and substance use [39, 40]. Hepatitis C co‐infection has been associated with suicide risk in PWH [41]. Hepatitis C cure is likely to reduce depressive symptoms [42], including among people who inject drugs [43], and those co‐infected with HIV [44], and has been associated with improvements in both health‐related quality of life and symptoms [45, 46, 47]. However, individuals cured of hepatitis C do not always report meaningful pain reduction [48, 49], and depressive symptoms may remain common among PWH following hepatitis C cure [50]. Post‐cure pain persistence is not fully understood. It may be related to unresolved HCV‐related neuropathic pain [51] or result from the impact of confounding factors such as psychiatric comorbidities [48]. PWH cured of hepatitis C may therefore be at particularly high risk of SI.

This study aimed to assess the relationship between self‐reported disabling physical pain and SI in aging HCV‐cured PWH, with a long‐standing history of hepatitis C virus (HCV) co‐infection, while accounting for other correlates or confounders such as depression and substance use.

## MATERIALS AND METHODS

### Design

We used data from a multi‐center cross‐sectional survey conducted between February 2018 and May 2019, and nested within the ANRS CO13 HEPAVIH French prospective cohort of adult (age ≥ 18 years) people living with HIV and HCV [50]. This survey aimed to assess changes in socio‐behavioural patient‐reported outcomes following HCV cure [52]. All participants enrolled in the cohort provided written informed consent. The ANRS CO13 HEPAVIH cohort was designed and conducted in accordance with the Declaration of Helsinki and received approval from the ethics committee of Cochin University Hospital in Paris. All cohort participants were invited to take part in the cross‐sectional survey.

### Data collection

Survey participants were invited to complete a paper self‐administered questionnaire collecting sociodemographic, behavioural, and perceived health‐related data. Among behavioural data, tobacco use was assessed with the following question: *‘Currently, are you a smoker?’* (No, and I never smoked; No, but I smoked in the past; Yes). Cannabis use over the past 12 months was also recorded (No; Less than once a month; One to three times a month; One to six times a week; Every day). Alcohol use was assessed using the AUDIT‐C screening tool [53]. Use of cocaine, crack, amphetamines, heroin, street buprenorphine, cathinones, ecstasy, and LSD or other hallucinogens in the past 4 weeks was collected (Never; Sometimes; Regularly; Daily). Regarding perceived health, participants completed the Patient Health Questionnaire‐9 (PHQ‐9) [54], a nine‐item symptom checklist suitable for use in routine HIV care to evaluate the presence and severity of depressive symptoms [55]. In addition, participants were asked whether they were currently taking a treatment against depression prescribed by a physician. Participants also completed the WHOQOL‐HIV BREF questionnaire, a 31‐item instrument developed to assess quality of life among PWH [56]. Biomedical characteristics were obtained from clinical data collected during the follow‐up visit closest to the date of questionnaire completion.

### Study population

From participants who completed the survey, we selected people who reported being cured of hepatitis C and with available data for SI. No age‐based criterion was further applied.

### Assessment of suicidal ideation

SI was assessed using the ninth item of the PHQ‐9: *‘Over the last 2 weeks, how often have you been bothered by thoughts that you would be better off dead or of hurting yourself in some way?’* with possible answers scored as 0 (never), 1 (several days), 2 (more than half the days), and 3 (nearly every day). Based on previous studies [57, 58], a score >0 was indicative of SI.

### Assessment of perceived disabling physical pain

Participants were asked the following question: ‘*To what extent do you feel that physical pain prevents you from doing what you need to do?’* (not at all; a little; a moderate amount; very much; an extreme amount) [56]. Participants who answered ‘very much’ or ‘an extreme amount’ were considered to be reporting disabling physical pain. Participants therefore reported the impact of pain on their daily functioning, but not the type of pain they experienced.

### Other potential correlates of suicidal ideation

Socio‐demographic characteristics examined for their association with SI included sex, age, place of birth (France vs. abroad), and living in a couple (yes; no). Being born abroad is associated with a lower standard of living in France [59], and it may be associated with poorer health status and detrimental health events [60, 61, 62], including mental health issues such as depression [63]. Living in a couple is likely to enhance social support and prevent from the occurrence of depression [64], and being unmarried has been associated with SI in PWH [17].

Depression was defined as either being prescribed an anti‐depressant treatment or, in cases of missing anti‐depressant treatment data, having a PHQ‐9 score ≥ 10. The social relationships dimension of quality of life was assessed using the corresponding domain of the WHOQOL‐HIV BREF questionnaire. This domain comprises four items, yielding a score ranging from 4 to 20, with higher scores indicating better social relationships. If only one of the four items was missing, its value was imputed using the mean of the remaining three items.

We used a substance use variable designed based on the approach used by Satre et al. [57]. Seven substances or substance groups were included: tobacco, cannabis, alcohol, stimulants (cocaine, crack, amphetamines), opioids (heroin, street buprenorphine), empathogens (cathinones, ecstasy), and LSD or other hallucinogens. For each of these, a binary score (0 or 1) was assigned to participants as follows: for tobacco use, in case of current use; for cannabis use, in case of a daily use; for alcohol use, in case of an AUDIT‐C score ≥3 for women and ≥4 for men; and for other substances, in case of any use in the past 4 weeks. The seven binary scores obtained were then summed to obtain a global substance use score.

The different approaches to score tobacco, cannabis, and alcohol use (based on a frequency of use) and other substance use (based on any use in the past 4 weeks) relied on the following considerations: (i) because the use of other substances is less common, frequency data were not collected, as statistical power would have been insufficient to account for it; (ii) published literature suggests dose–response relationships between the use of tobacco [65], cannabis [66], alcohol [67], and suicidal behaviours.

Biomedical characteristics included self‐reported HIV transmission mode (male‐to‐male sex; heterosexual sex; injecting drug use; other/unknown), HIV viral load (undetectable; detectable; no lab results), time since HIV and time since HCV diagnosis (in years), CD4 cell count (>500; 351–500; 200–350; <200, in cells/mm^3^), and history of treatment with efavirenz or interferon.

### Statistical analyses

Characteristics of the study population were described and compared according to the presence of SI, using the Chi‐squared test or Fisher exact test for categorical variables and the Kruskal–Walli's test for continuous variables.

The association between perceived disabling physical pain and SI was explored using multivariable logistic regression. Explanatory variables were first tested in univariable analyses. Those with a *p*‐value <0.2 were eligible for the multivariable analysis [68]. A backward stepwise selection procedure was used to select variables for the final multivariable model, with a *p*‐value threshold of 0.05. Variables eligible for multivariable analyses which were eliminated during the backward stepwise selection procedure were then reintroduced to test for potential changes in the significance level of associations and/or changes in the adjusted odds ratio (aOR) estimates of variables in the final model. If the aOR for at least one of the remaining variables changed by more than 25%, the eliminated variable was deemed to be a confounder and restored to the model [69]. A robust sandwich estimator was used to estimate the variance of the odds ratios. All analyses were performed with Stata software version 17.0 for Windows (StataCorp LP, College Station, TX).

## RESULTS

### Study population characteristics

Characteristics of the 396 participants in the study population are presented in Table 1. The majority were male (73.2%), and the median [interquartile range, IQR] age was 55.5 [53–59] years. The median [IQR] time since HIV diagnosis was 29.0 [23.3–31.8] years, and the median [IQR] time since HCV diagnosis was 21.2 [16.9–25.0] years. Sixty‐one percent of participants acquired HIV via injection drug use (21.0% of them reported SI). SI was reported by 70 participants (17.7%). Approximately one‐third of participants (32.1%) reported using one substance; among them, 74.0% smoked tobacco and 25.6% had unhealthy alcohol use. Another third (34.3%) reported using at least two substances, with the vast majority being tobacco smokers (94.2% of those using two substances and 93.9% of those using three or more). Perceived disabling physical pain was reported by 46 participants (11.9%), including 18 who also reported SI (Table 1). Among participants with SI, 48.6% reported using at least two substances, 44.3% had depression, and 17.1% had a detectable HIV viral load.

**TABLE 1:** Study population characteristics according to suicidal ideation (cross‐sectional survey nested within the ANRS CO13 HEPAVIH cohort, *N* = 396).

Characteristics (% of missing data)	Study population (*N* = 396)	No suicidal ideation (*N* = 326)	Suicidal ideation (*N* = 70)	*p*‐Value^a^
	*N* (%) or median [IQR]	*N* (%) or median [IQR]	*N* (%) or median [IQR]	
Perceived disabling physical pain^b^ (2.0)				<0.001
No	342 (88.1)	292 (91.3)	50 (73.5)	
Yes	46 (11.9)	28 (8.7)	18 (26.5)	
Sex (0)				0.205
Male	290 (73.2)	243 (74.5)	47 (67.1)	
Female	106 (26.8)	83 (25.5)	23 (32.9)	
Age (in years) (0)	55.5 [53–59]	55.5 [53–59]	55.5 [52–59]	0.740
Place of birth (0)				0.389
France	307 (77.5)	250 (76.7)	57 (81.4)	
Abroad	89 (22.5)	76 (23.3)	13 (18.6)	
Living in a couple (1.0)				0.090
No	208 (53.1)	165 (51.1)	43 (62.3)	
Yes	184 (46.9)	158 (48.9)	26 (37.7)	
HIV transmission mode (0)				0.121
Male‐to‐male sex	44 (11.1)	37 (11.3)	7 (10.0)	
Heterosexual sex	59 (14.9)	54 (16.6)	5 (7.1)	
Injecting drug use	243 (61.4)	192 (58.9)	51 (72.9)	
Other/unknown	50 (12.6)	43 (13.2)	7 (10.0)	
HIV viral load^c^ (0)				0.049
Undetectable	326 (82.3)	275 (84.4)	51 (72.9)	
Detectable	39 (9.8)	27 (8.3)	12 (17.1)	
No lab results	31 (7.8)	24 (7.4)	7 (10.0)	
Time since HIV diagnosis (in years) (0.3)	29.0 [23.3–31.8]	29.1 [23.3–31.9]	28.6 [23.4–31.3]	0.545
Time since HCV diagnosis (in years) (3.5)	21.2 [16.9–25.0]	21.1 [16.8–34.8]	22.0 [17.0–25.8]	0.375
CD4 cell count (in cells/mL) (0)				0.177
>500	242 (61.1)	204 (62.6)	38 (54.3)	
351–500	59 (14.9)	42 (12.9)	17 (24.3)	
200–350	38 (9.6)	31 (9.5)	7 (10.0)	
<200	16 (4.0)	13 (4.0)	3 (4.3)	
No lab results	41 (10.4)	36 (11.0)	5 (7.1)	
History of interferon treatment (2.8)				0.146
No	117 (30.4)	92 (28.8)	25 (37.9)	
Yes	268 (69.6)	227 (71.2)	41 (62.1)	
History of efavirenz treatment (2.8)				0.174
No	210 (54.5)	169 (53.0)	41 (62.1)	
Yes	175 (45.5)	150 (47.0)	25 (37.9)	
Number of psychoactive substances used^d^ (0)				0.007
0	133 (33.6)	121 (37.1)	12 (17.1)	
1	127 (32.1)	103 (31.6)	24 (34.3)	
2	103 (26.0)	78 (23.9)	25 (35.7)	
≥3	33 (8.3)	24 (7.4)	9 (12.9)	
Current tobacco use (0)				0.010
No	174 (43.9)	153 (46.9)	21 (30.0)	
Yes	222 (56.1)	173 (53.1)	49 (70.0)	
Unhealthy alcohol use^e^ (1.3)				0.065
No	255 (65.2)	216 (67.3)	39 (55.7)	
Yes	136 (34.8)	105 (32.7)	31 (44.3)	
Daily cannabis use (1.8)				0.128
No	343 (88.2)	385 (89.3)	58 (82.9)	
Yes	46 (11.8)	34 (10.7)	12 (17.1)	
Use of other psychoactive substances^f^ (6.6)				0.226
No	344 (93.0)	284 (93.7)	60 (89.5)	
Yes	26 (7.0)	19 (6.3)	7 (10.5)	
Depression^g^ (6.1)				<0.001
No	305 (82.0)	266 (88.1)	39 (55.7)	
Yes	67 (18.0)	36 (11.9)	31 (44.3)	
Quality of life related to social relationships^h^ (3.0)	14 [12–16]	15 [13–7]	11 [9–13]	<0.001

Abbreviations: HCV, hepatitis C virus; IQR, interquartile range.

^a^ Chi‐squared or Fisher exact test for categorical variables, and Kruskal‐Wallis test for continuous variables.

^b^ Answering ‘very much’ or ‘an extreme amount’ to the WHOQOL‐HIV BREF item ‘To what extent do you feel that physical pain prevents you from doing what you need to do?’ [56].

^c^ According to the laboratory test threshold.

^d^ Seven substances or substance groups were considered, as follows: tobacco, cannabis, alcohol, stimulants, opioids, empathogens, and LSD or other hallucinogens. For each of them, a score of 0 or 1 was assigned to each participant according to criteria detailed in the text. The global substance use score is the sum of those scores.

^e^ Unhealthy alcohol use is defined as an AUDIT‐C score ≥3 for women and ≥4 for men [53].

^f^ Any use of stimulants, opioids, empathogens, and LSD or other hallucinogens in the past 4 weeks.

^g^ Depression was defined as either being prescribed an anti‐depressant treatment or, for the 23 participants (5.8% of the study sample) with missing data on anti‐depressant treatment prescription, having a PHQ‐9 score ≥10.

^h^ The social relationships dimension of quality of life was assessed through the corresponding WHOQOL‐HIV BREF domain [56]. The associated score ranges from 4 to 20, with higher score values denoting better social relationships.

### Perceived disabling physical pain and suicidal ideation: a multivariable analysis

After multivariable adjustment, participants reporting disabling physical pain had a three‐fold higher risk of SI (aOR [95% confidence interval]: 3.07 [1.29–7.34], *p* = 0.012) (Table 2). Moreover, the use of at least two substances (2.78 [1.07–7.26] and 5.42 [1.73–16.93], for two substances and three or more substances, respectively), depression (5.52 [2.66–11.43]), and a lower quality of life related to social relationships (0.72 [0.64–0.80]) were also significantly associated with SI.

**TABLE 2:** Association between perceived disabling physical pain and suicidal ideation in people living with HIV cured of hepatitis C (univariable and multivariable logistic regression, cross‐sectional survey nested within the ANRS CO13 HEPAVIH cohort).

Explanatory variables	Univariable analysis (*N* = 396)	*p*‐Value	Multivariable analysis (*N* = 376)	*p*‐Value
	OR [95% CI]		aOR [95% CI]	
Perceived disabling physical pain^a^				
No (ref.)	1		1	
Yes	3.75 [1.93–7.30]	<0.001	3.07 [1.29–7.34]	0.012
Sex				
Male (ref.)	1			
Female	1.43 [0.82–2.50]	0.207		
Age (in years)	0.98 [0.9–1.02]	0.303		
Place of birth				
France (ref.)	1			
Abroad	0.75 [0.39–1.45]	0.390		
Living in couple				
No (ref.)	1			
Yes	0.63 [0.37–1.08]	0.092		
HIV transmission mode		**0.138**		
Male‐to‐male sex	2.04 [0.60–6.94]	0.252		
Heterosexual sex (ref.)	1			
Injecting drug use	2.87 [1.09–7.55]	0.033		
Other/unknown	1.76 [0.52–5.93]	0.364		
HIV viral load^b^		**0.056**		
Undetectable (ref.)	1			
Detectable	2.40 [1.14–5.04]	0.021		
No lab results	1.57 [0.64–3.85]	0.321		
Time since HIV diagnosis (in years)	0.99 [0.96–1.03]	0.672		
Time since HCV diagnosis (in years)	1.01 [0.96–1.05]	0.779		
CD4 cell count (in cells/mL)		**0.177**		
>500 (ref.)	1			
351–500	2.17 [1.12–4.21]	0.022		
200–350	1.21 [0.50–2.96]	0.672		
<200	1.24 [0.34–4.56]	0.747		
No lab results	0.75 [0.27–2.02]	0.565		
History of interferon treatment				
No (ref.)	1			
Yes	0.66 [0.38–1.16]	0.148		
History of efavirenz treatment				
No (ref.)	1			
Yes	0.69 [0.40–1.18]	0.177		
Number of psychoactive substances used^c^		**0.009**		**0.027**
0 (ref.)	1		1	
1	2.39 [1.14–5.02]	0.021	2.02 [0.79–5.18]	0.143
2	3.25 [1.55–6.87]	0.002	2.78 [1.07–7.26]	0.037
≥3	3.81 [1.45–10.06]	0.007	5.42 [1.73–16.93]	0.004
Depression^d^				
No (ref.)	1		1	
Yes	5.81 [3.26–10.36]	<0.001	5.52 [2.66–11.43]	<0.001
Quality of life related to social relationships^e^	0.70 [0.64–0.80]	<0.001	0.72 [0.64–0.80]	<0.001

*Note:* Bold values in the “*p*‐Value” column corresponds to the overall *p*‐values for the explanatory variables having more than 2 categories.

Abbreviations: aOR, adjusted odds ratio; CI, confidence interval; OR, odds ratio.

^a^ Answering ‘very much’ or ‘an extreme amount’ to the WHOQOL‐HIV BREF item ‘To what extent do you feel that physical pain prevents you from doing what you need to do?’ [56].

^b^ According to the laboratory test threshold.

^c^ Seven substances or substance groups were considered, as follows: tobacco, cannabis, alcohol, stimulants, opioids, empathogens, and LSD or other hallucinogens. For each of them, a score of 0 or 1 was assigned to each participant according to criteria detailed in the text. The global substance use score is the sum of those scores.

^d^ Depression was defined as either being prescribed an anti‐depressant treatment or, for the 23 participants (5.8% of the study sample) with missing data on anti‐depressant treatment prescription, having a PHQ‐9 score ≥10.

^e^ The social relationships dimension of quality of life was assessed through the corresponding WHOQOL‐HIV BREF domain [56]. The associated score ranges from 4 to 20, with higher score values denoting better social relationships.

## DISCUSSION

Our findings confirm the hypothesis that in aging PWH cured of HCV, perceived disabling physical pain is strongly associated with suicidal ideation, even when accounting for other known predictors, including depression and substance use. They also confirm that lower social relationship‐related quality of life is independently associated with SI.

Associations between SI and depression, substance use, and low social support have previously been reported among PWH [13]. Overall quality of life has also been associated with SI in this population [13]. In a representative study of PWH receiving care in France, suicide risk has been found associated with reporting a feeling of loneliness [41], consistent with the results found in the present study, and suggesting the importance of social support interventions in preventing suicide [70].

However, to our knowledge, no previous study has specifically documented an independent association between physical pain and SI among PWH. Sherr et al. found such an association between physical burden score and SI in PWH in the UK [71]. The association between perceived disabling physical pain and SI we found aligns with findings reported in individuals experiencing physical pain [72], older adults [73], and in those with chronic pain in the general population [74, 75]. In the latter group, independent and concomitant effects of perceived pain and depression on suicide behaviours have been reported [76].

It should be noted that pain and depression are closely interrelated and may mutually exacerbate both physical and psychological symptoms [77]. Pain can also manifest as a symptom of depression in the absence of any nociceptive stimulus [78]. Therefore, we cannot exclude the possibility that our measure of pain partially captured depressive symptoms, and that part of the observed effect of pain may, in fact, be attributable to depression.

As previously reported [13], depression had the strongest association with SI among all variables examined in our study, underscoring the importance of depression screening and referral to care to reduce suicide risk among PWH. In our study population, the risk of SI was more than three times higher among participants reporting perceived disabling physical pain compared to those who did not. Those results highlight the potential clinical utility of brief pain screening [79], alongside depression and substance use in PWH, particularly among those with a history of chronic hepatitis C. Notably, substance use may serve as a pain self‐management strategy among PWH [80], and chronic pain in PWH is more prevalent in older individuals with complex medical, psychiatric, and substance use comorbidities [81]. Additionally, individuals with chronic opioid exposure, such as individuals with a history of injecting drug use (61.4% of our study population), may develop hyperalgesia, defined as an increased sensitivity and experience of pain [82, 83]. In a previous French cohort, PWH who had been infected via injecting drug use reported more painful anti‐retroviral‐related side effects than those who had been infected via other ways [84].

Consistent with previous studies, we observed an association between substance use—especially polysubstance use—and SI [18, 57, 85, 86, 87, 88]. It has been shown that people with and without a history of substance use both experience great benefits from direct‐acting anti‐viral treatments, in terms of mortality and liver‐related events [89, 90]. However, even if decreases may be observed, substance use is likely to persist after HCV cure [52, 91, 92]. Therefore, integrated HCV treatment models that include addiction care [93, 94, 95] may, if effective in reducing substance use, lower the risk of SI among individuals previously infected with HCV.

The prevalence of SI was high (17.8%), in line with in the findings of Pei et al.'s meta‐analysis among PWH (20.9%) [12]. These findings suggest that a history of HCV infection does not further increase the risk of SI in this population. This figure is, however, higher than the suicidality prevalence of 3.96% estimated in the European general population [96]. Although direct comparisons are difficult, our prevalence estimate also appears to lie towards the upper end of the range reported in studies focusing on aging populations [73, 97, 98, 99, 100].

The main strength of our study lies in the simultaneous inclusion of data on depression, substance use, physical pain, the social dimension of quality of life, and clinical data in the analysis of factors associated with SI. This approach enabled us to highlight the independent contribution of perceived disabling physical pain on SI. SI may arise from a great variety of conditions. While pain seems to be a major driver of the relationship between multi‐morbidity and SI [101], the complexity of SI calls on us not to overlook the possible influence of unmeasured confounding factors, including comorbid conditions. Other mental health conditions likely to impact SI, such as post‐traumatic stress disorder [102], as well as the use of non‐pharmacological therapies, such as psychotherapy, were also not assessed. We used a single item to assess physical pain. In return for its simplicity of implementation, such a tool may have masked the role of diverse types of pain. It may also be biased by inter‐individual differences in perceptions of pain, and by the diversity of daily needs that may lead to different levels of reported disability for a given level of pain. Further research is needed to identify which types, location, and dimensions of physical pain should be prioritized for management in PWH cured of HCV. Similarly, substance use and receiving drug prescriptions against depression were self‐reported, and may have been underreported. Lastly, we were limited by the cross‐sectional nature of data collection, which did not enable us to assess how SI and associated factors evolve over time, hindering the possibility of inferring any causal directions in the associations we found.

In conclusion, these findings highlight that disabling physical pain should be systematically addressed among PWH cured of HCV, given its independent association with SI. Routine HIV follow‐up care should integrate systematic screening for pain, mental health problems, and lack of social support. Timely referral to specialized services may help prevent future suicidal behaviours in this population.

## AUTHOR CONTRIBUTIONS

PS, KO, TS, SA, SB‐R contributed to the study conception, design, data acquisition and interpretation. CR performed formal analysis. TB, CR, CP, PC and FM were involved in study conception, methodology, interpretation and drafting of the manuscript. All authors reviewed and approved the final version of the manuscript.

## FUNDING INFORMATION

This work was supported by the ANRS Emerging Infectious Diseases, with the participation of SIDACTION, Abbott France, Glaxo‐Smith‐Kline, Roche, Schering‐Plough, BMS, and Merck‐Serono.

## CONFLICT OF INTEREST STATEMENT

The authors declare no conflicts of interest.

## ETHICS STATEMENT

The ANRS CO13 HEPAVIH cohort was designed and implemented in accordance with the Declaration of Helsinki and was approved by the ethics committee of the Cochin University Hospital in Paris.

## PATIENT CONSENT STATEMENT

Written informed consent was collected for all participants.

## Data Availability

The data that support the findings of this study are available on request from the corresponding author. The data are not publicly available due to privacy or ethical restrictions.
